# Supplementary material for: The interactome of intact mitochondria by cross-linking mass spectrometry provides evidence for coexisting respiratory supercomplexes
Source: Mol Cell Proteomics. 2017 Dec 8;17(2):216–32. doi: 10.1074/mcp.RA117.000470 (PMC5795388; doi:10.1074/mcp.RA117.000470)
Supplement: Supplemental Data [file supp_RA117.000470_133922_0_supp_23978_fzffwf.zip › spectra_annotation/mito_spectra_annotation/42-1-8-1-8-1.pdf]

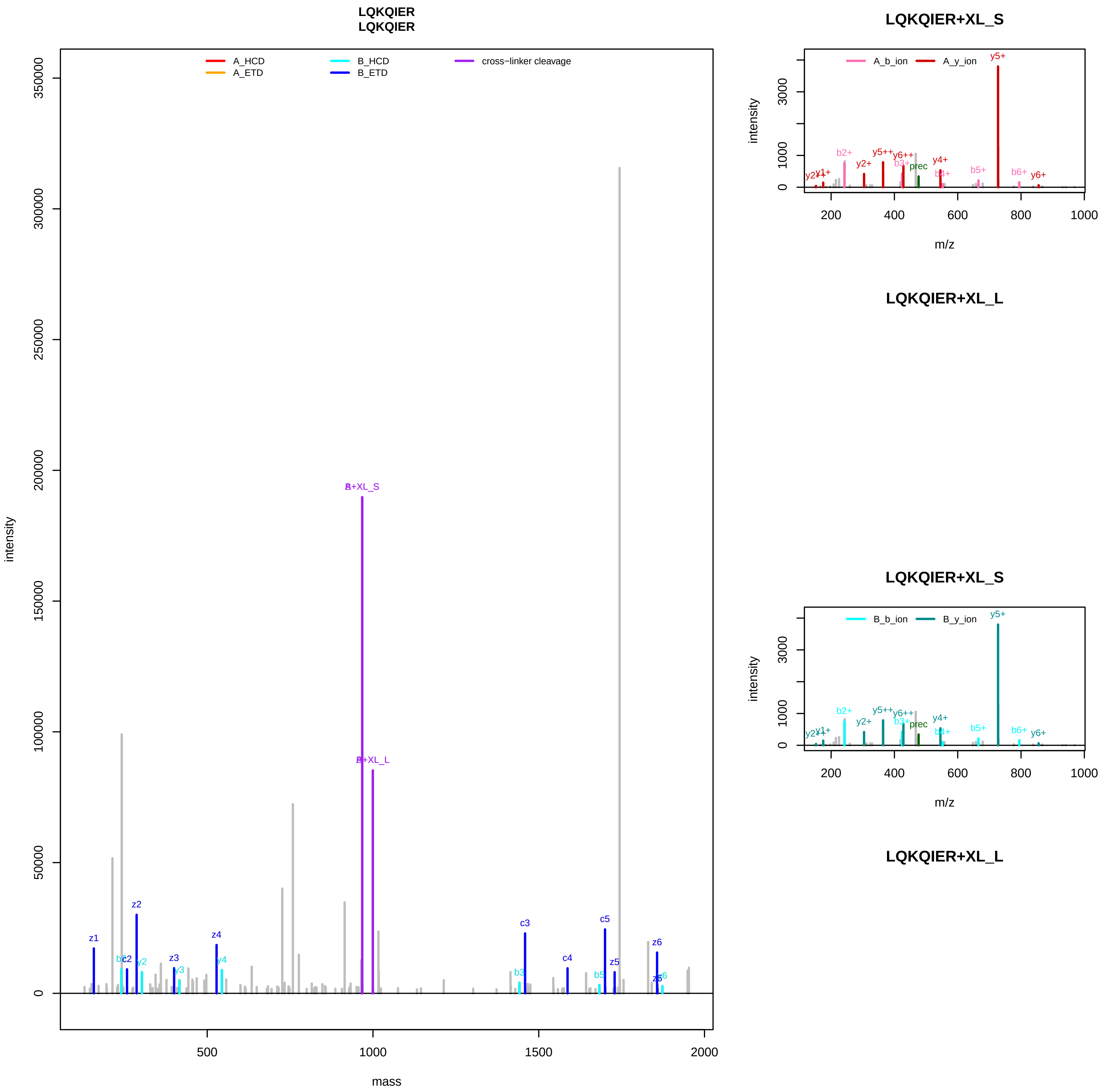

LQKQIER
LQKQIER
A_HCD
A_ETD
B_HCD
B_ETD
cross−linker cleavage
A+XL_S
A+XL_L
z1
z2
c2
y2
z3
y3
z4
y4
c3
b3
c4
c5
b5
z5
z6
intensity
mass
LQKQIER+XL_S
A_b_ion
A_y_ion
y5+
b2+
y5++
y6++
y2+
b3+
prec
y4+
b4+
b5+
b6+
y6+
y1+
intensity
m/z
LQKQIER+XL_L
LQKQIER+XL_S
B_b_ion
B_y_ion
intensity
m/z
LQKQIER+XL_L
